# Clinical, Genetic, and Prognostic Features of Adrenocortical Tumors in Children: A 10-Year Single-Center Experience

**DOI:** 10.3389/fonc.2020.554388

**Published:** 2020-10-15

**Authors:** Evelina Miele, Angela Di Giannatale, Alessandro Crocoli, Raffaele Cozza, Annalisa Serra, Aurora Castellano, Antonella Cacchione, Maria Giuseppina Cefalo, Rita Alaggio, Maria Debora De Pasquale

**Affiliations:** ^1^Department of Paediatric Haematology/Oncology Cell and Gene Therapy, Bambino Gesù Children's Hospital, IRCCS, Rome, Italy; ^2^Department of Surgery, Bambino Gesù Children's Hospital, IRCCS, Rome, Italy; ^3^Department of Laboratories, Pathology Unit, Bambino Gesù Children's Hospital, IRCCS, Rome, Italy

**Keywords:** adrenocortical tumors, children, Li-Fraumeni Syndrome, Beckwith–Wiedeman syndrome, mitotane, immunotherapy, targeted therapies, prognosis

## Abstract

**Background and Aims:** Pediatric adrenocortical tumors (ACTs) are very rare endocrine neoplasms in childhood. In this study, we performed a retrospective analysis of children with ACT treated at our institution by examining clinical and genetic disease features, treatment strategies, and outcomes.

**Methods:** We retrospectively analyzed a cohort of 13 children treated at the Bambino Gesù Children's Hospital from November 2010 to March 2020.

**Results:** The median age at diagnosis was 17 months (range = 0–82 months). The female: male ratio was 3.3/1. Mixed symptomatology (>1 hormone abnormality) was the most common presentation (46.1%). In three cases, the tumor was detected during prenatal or perinatal echographic screening. All patients presented with localized disease at diagnosis and underwent total adrenalectomy. Six patients were identified as having malignancies according to the Wieneke scoring system, five benign, and two undetermined. Seven patients underwent mitotane adjuvant therapy for 12 months. There was metastatic disease in three patients, with no correlation with age or Wieneke score. The most common sites of metastases were the liver and lungs. Metastatic patients were treated with surgery (*n* = 2), mitotane (*n* = 1), chemotherapy (*n* = 2) associated with anti-EGFR (*n* = 1), or immunotherapy with anti-PD1 (pembrolizumab) (*n* = 1); two patients achieved complete disease remission. Overall 2- and 5-year survival rates were 100%, with a median follow-up of 5 years (range = 2–9.5 years). Two- and 5-year disease free survival was 76.9 and 84.6%, respectively (95% confidence interval = −66.78–114.76 months). All patients are alive, 12 without disease, and one with stable disease. Genetic analyses showed TP53 germline mutations in six of eight patients analyzed (five inherited, one *de novo*). One patient had Beckwith–Wiedemann syndrome, with mosaic paternal uniparental disomy of chromosome 11, in both neoplastic and healthy adrenal tissue.

**Conclusion:** We report the cases of 13 patients treated for ACT, including 12 aged <4 years at diagnosis, with a relative short time from symptoms onset. Our cohort experienced an excellent prognosis. TP53 mutation was found in 75% of tested patients (6/8) confirming the need to perform genetic tests and familial counseling in this disease.

## Introduction

Pediatric adrenocortical tumors (ACTs) include both benign adrenocortical adenomas (ACA) and highly aggressive adrenocortical carcinomas (ACC). They are very rare neoplasms of childhood, with a reported incidence of just 0.2–0.3 new cases per 1 million children per year (1, 2) and accounting for 6% of all adrenal cancers in children (3). ACC incidence rises of 10–15 times the worldwide rate in Southern Brazil, which is likely associated with high prevalence of the founder p.R337H TP53 mutation (4). ACT can occur in the context of several cancer predisposition syndromes; in fact, most childhood ACC are linked to genetic susceptibility, although their pathogenesis is not completely understood (5). Prognosis of pediatric ACT patients is highly heterogeneous and hardly predictable in clinical practice. There is considerable variability in clinical presentation, from tumors with an indolent clinical course to highly malignant tumors with dismal prognosis. Risk factors for poor outcomes in patients with ACT include older age, higher mitotic rate, higher percent of necrosis, and larger tumor size (3). In some cases, a delayed diagnosis may contribute to advanced stages and poor prognosis in these patients (3).

Pediatric ACC patients generally have overall 5-year survival ranging from 30 to 70%, depending on disease presentation (6–8). Despite multimodal therapeutic approaches, outcomes remain poor in patients with metastatic disease, with an estimated 5-year survival <20% (1, 2, 7, 9–11). No effective therapy is currently available for advanced and metastatic ACC; the only treatment leading to cure and long-term survival remains complete surgical resection (6, 7). Adjuvant mitotane, chemotherapy, and/or radiotherapy may reduce recurrence. Arterial chemoembolization, radiotherapy, and radiofrequency ablation are treatment options reported in cases of advanced disease in adulthood (2, 12, 13). However, because many children with ACT carry germline TP53 mutations, radiation therapy in pediatric ACTs has not been studied and should be avoided (6, 14). On the other hand, ACA histology is associated with excellent prognosis, but only about 20% of pediatric ACTs are identified as ACA, and the correct distinction between adenoma and carcinoma is difficult (15). Indeed, there are no well-defined pathological malignancy criteria for pediatric ACT, whereas adult tumors can be classified based on Weiss or Van Slooten criteria (16, 17). The Wieneke criteria, considering tumor size, local invasion, and histological features, are reported useful in discriminating benign from malignant tumors and predicting the prognosis of pediatric ACT (11, 18).

In the present study, we performed a retrospective analysis examining clinical and genetic disease features, treatment strategies, and outcomes in children with ACT in a single institution.

## Methods

We retrospectively reviewed medical records of children affected by ACT and admitted to our hospital between November 2010 and March 2020. All patients included in this study were <18 years old with ACT confirmed by pathological review. The following data were collected: general clinical features (gender, age, clinical symptoms, and signs), imaging, pathological characteristics, and prognosis.

Given our interest in examining genetic factors in this disease, *TP53* mutations analysis was performed on peripheral blood DNA samples from the patients and their parents, by using BigDye direct Sanger sequencing of exons 2–11 and intron–exon boundaries of polymerase chain reaction products by an ABI automated sequencer (Applied Biosystems, Foster City, CA). Gene dosage was evaluated by multiplex ligation-dependent probe amplification (MLPA) using the MRC-Holland SALSA MLPA PO56 TP53 probe set (MRC-Holland, Amsterdam, the Netherlands) according to the manufacturer's instructions. Chromosome microarray analysis was performed in patients 1 (blood sample) and 9 (blood, saliva, skin fibroblasts, healthy and neoplastic adrenal samples) by using SNP- array (single-nucleotide polymorphism array) on platform CytoSNP-850K BeadChip (Illumina, San Diego, CA) with an average resolution of 100 Kb. Outcomes were reported as alive with no evidence of disease, alive with evidence of disease, and dead of disease. The Wieneke index was applied for diagnosis and prognosis definition.

## Results

This retrospective cohort included 13 children. Median age at pathological diagnosis was 17 months (range = 0–82 months). Female-to-male ratio was 3.3/1 (Table 1). Mixed symptomatology (>1 hormone abnormality) was the most common presentation (46.1%, *n* = 6), (Table 1). In three cases (patients 5, 7, and 13), diagnosis was performed in asymptomatic patients via prenatal (patient 13) or perinatal echographic screening for congenital dysplasia of the hip (patients 5 and 7). All patients presented with localized disease at diagnosis and underwent total adrenalectomy by laparotomy (*n* = 12) or laparoscopic surgery (*n* = 1, patient 13; Table 2). The Wieneke score system was applied for diagnosis and prognosis definition: six patients were assigned to the malignant category, five to the benign category, and two had a diagnosis of tumor with uncertain biological behavior (indeterminate), (Table 3).

**Table 1:** Clinical features at presentation of 13 pediatric patients with adrenocortical tumors.

**Clinical feature**	**All patients (*n* = 13)**	**Age <24 months (*n* = 8)**	**Age ≥24 months (*n* = 5)**
**Age at onset of symptoms, months**			
Median	17	5.5	39
Range	0–82	0–22	24–82
**Sex**, ***n***			
Male	3	2	1
Female	10	6	4
Female:male ratio	3.3:1	3:1	4:1
**Type of presentation**, ***n***			
Virilization only^a^	3 (23.1%)	2	1
Cushing syndrome only	0	0	0
Hypertension only	0	0	0
Mixed tumor	6 (46.1%)	3	3
Asymptomatic	3 (23.1%)	3	0
Unknown^b^	1 (7.7%)	0	1
**Duration of symptoms, months**			
Median	1	0.5	1
Range	0–10	0–10	1–3

a Indicated by clinical and/or laboratory evidence of abnormal production of more than one hormone,

b *The patient was diagnosed at another institution, and the initial medical records were not available*.

**Table 2:** Therapeutic approach.

**Surgery/no. of patients**	**Adjuvant mitotane/no. of patients**	**Chemotherapy/no. of patients**	**Immunotherapy/no. of patients**	**Outcome/no. of patients**
LTUA/8	No/6	No/11	No/11	CR/12
LTUA + linfoadenectomy/3	Yes/7	Yes/2^*^^∧^	Yes/2°	SD/1^•^
LTUA + bioptic sampling/1				
LUA/1				

LTUA, laparotomic unilateral adrenalectomy; LUA, laparoscopic unilateral adrenalectomy.

* Patient 9 received cisplatin (40 mg/mq) days 1 and 9, doxorubicin (20 mg/mq) days 1 and 8, etoposide (100 mg/mq) days 5–7;

∧ patient 8 received after relapse vincristine/irinotecan/panitumumab and then gemcitabine/oxaliplatin/panitumumab; °patients 8 and 11 received pembrolizumab;

• *patient 8*.

**Table 3:** Pathological features in childhood ACT.

**#**	**Tumor size**	**Growth pattern**	**Ki67 (%)**	**Atypical mitosis**	**Nuclear pleomorphism**	**Necrosis**	**Capsular invasion**	**Vascular invasion**	**N+**	**M+**	**Other**	**Wieneke score**
1	77 g	Diffuse	2–8	No	No	Yes	No	Yes	No	No	Reticolinic pattern anomalies	Benign
	6 × 5.5 × 4 cm				–							
2	20 g	Diffuse	5	Yes	Yes	No	Yes	No	No	No	p53 +++ nuclear	Intermediate
	2.5 × 2 × 1 cm				++							
3	100 g	Diffuse	30	Yes	Yes	Yes	No	Yes	No	No	p53 + 70%	Malignant
	8 × 6.5 × 5 cm				+++							
4	159 g	Solid	10–40	Yes	Yes	Yes	Yes	Yes	No	No	/	Malignant
	9 × 7 × 4.5 cm				+++							
5	40 g	Solid	5–10	No	+/–	No	No	Yes	No	No	p53 neg	Benign
	3.2 × 2.5 × 2 cm											
6	50 g	Solid	2	No	Yes	No	No	No	No	No	p53+ nuclear 10%	Benign
	2.5 × 2 × 3.5 cm				+							
7	10 g	Diffuse	20–30	Yes	Yes	Yes	Yes	No	No	No	p53 +	Intermediate
	3.5 × 3 × 1.5 cm				+++							
8	49.3 g	Solid	30–40	Yes	Yes	Yes	No	Yes	No	No	p53 +	Malignant
	5.5 × 4.5 × 4 cm				+++							
9	50 g	Diffuse	20–30	Yes	Yes	Yes	Yes	Yes	Yes	No	p53 +	Malignant
	6 × 5 × 4 cm				+++							
10	33 g	Solid	8	No	No	Yes, focal	No	No	No	No	p53 neg	Benign
	5 × 4 × 2.5 cm											
11^*^	NA	Solid	High	Yes	Yes	Yes	na	No	na	No	/	Malignant
					+++							
12	48 g	Solid	15–20	Yes	Yes	Yes	Yes	Yes	No	No	p53 –/+	Malignant
	5.5 × 4.5 × 4 cm				+++							
13	18 g	Diffuse	5–30	No	No	No	No	No	No	No	p53 +/–	Benign
	5 × 3.7 × 2.5 cm											

* *Diagnosis formulated in a different center. NA, not available; N+, nodal metastasis; M+, distant metastasis; p53+/–, positivity in <50% but more than 25% of cells; p53–/+, positivity in <25% of cells*.

**Figure 1:**
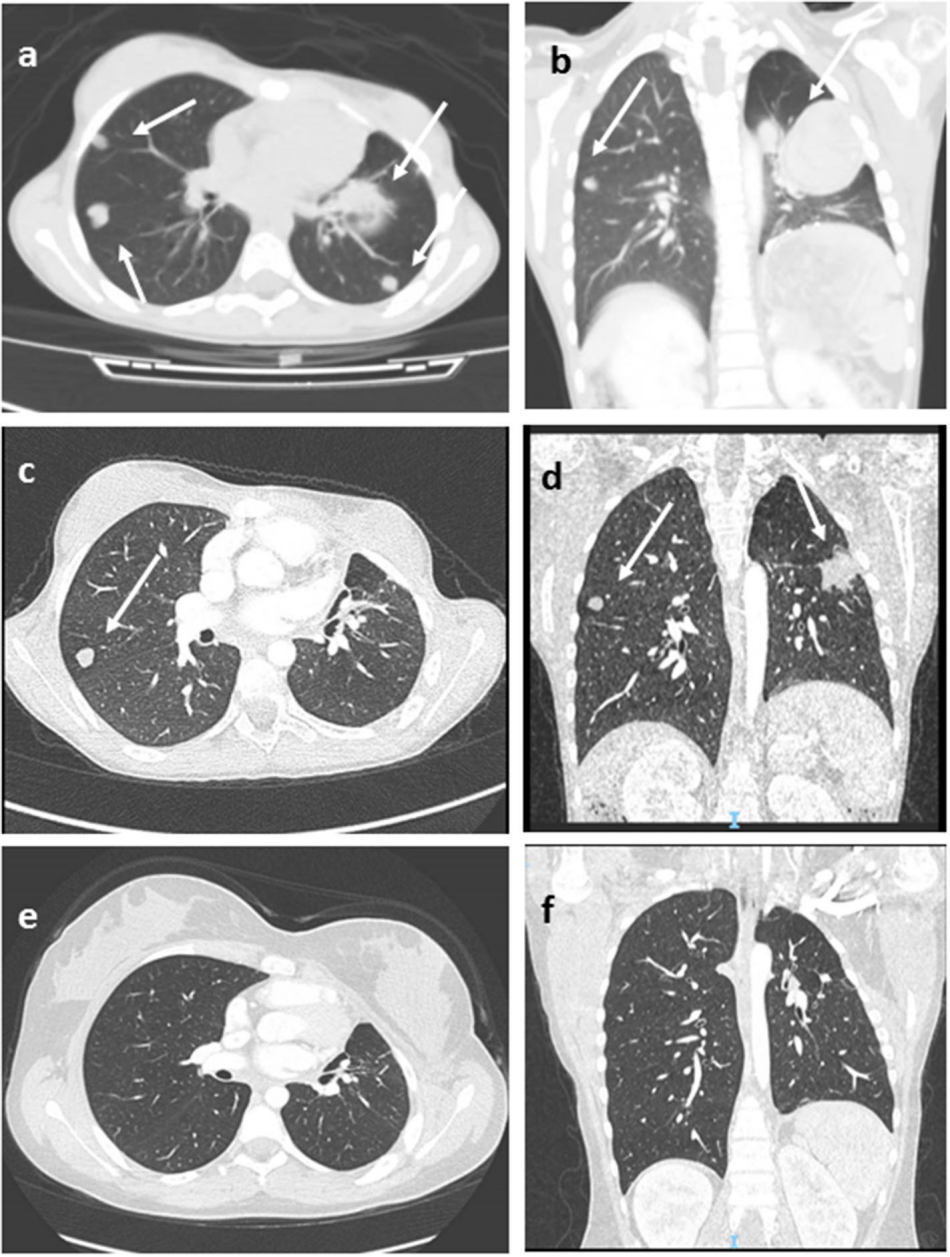
Lung Computed tomography (CT) images (patient #11) showing multiple metastatic lesions (arrows), before **(a,b)**, after 3 months of pembrolizumab therapy **(c,d)**, and at last follow-up **(e,f)** in complete disease remission. Axial **(a,c,e)**, coronal **(b,d,f)**.

Seven patients underwent mitotane-based adjuvant therapy for 12 months (Table 2). Metastatic disease appeared in three patients after 3, 18, and 42, months, respectively, in one case under treatment and in two during follow-up. No correlation with age or with Wieneke category was observed in metastatic/relapsed patients (the Fisher exact test was not significant). The most common sites of metastases were the liver and lungs. Relapsed and metastatic patients were treated with surgery (2 patients), mitotane (1 patient), chemotherapy (2 patients) associated or not with anti-EGFR (1 patient), or immunotherapy with anti-PD1 (pembrolizumab) (1 patient); two patients achieved complete disease remission (Figure 1). Overall 2- and 5-year survival rates were both 100%, with a median follow-up of 5 years (range = 2–9.5 years). Two- and 5-year disease-free survival was 76.9 and 84.6%, respectively (95% confidence interval = −66.78–114.76 months). At present, 12 patients are alive with no evidence of disease, and one is alive with evidence of metastatic disease.

**Figure 2:**
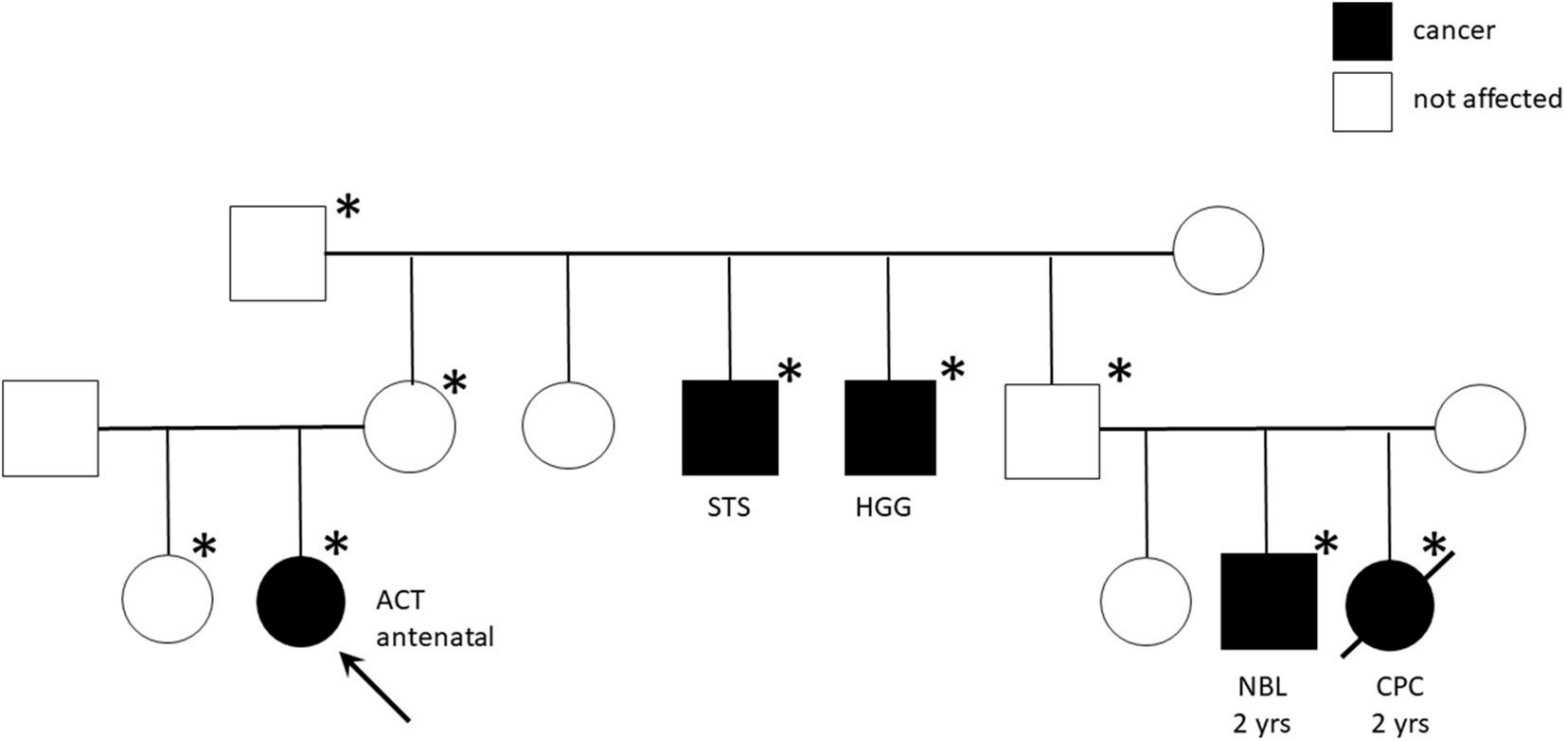
Pedigree chart of Li-Fraumeni syndrome with TP53 mutation c427G>A (pVal143Met). The arrow indicates the proband (Case #13). Squares represent males, circles represent females, and black symbols indicate individuals, ^*^indicates individuals carrying the mutation. ACT, adrenocortical tumor; STS, Soft Tissue Sarcoma; HGG, High Grade Glioma; NBL, Neuroblastoma; CPC, Choroid Plexus Carcinoma; yrs, years.

Genetic analyses were conducted for eight patients showing *TP53* germline mutations in six (five inherited and one *de novo*) (Table 4). The most part of detected mutations were already recognized as pathogenic. All the carrier parents were asymptomatic, but family history was positive for cancer in four patients (Table 4). In two cases, it was strongly suggestive of Li-Fraumeni syndrome (LFS) for the tumor histotypes (e.g., alveolar rhabdomyosarcomas, choroid plexus carcinoma) and the very young age of the affected individuals (Figure 2). One patient (patient 9) had Beckwith–Wiedemann syndrome (BWS) with clinical features (macrosomia, hyperinsulinism, hyperglycemia, and tumor) and paternal uniparental disomy of chromosome 11 on neoplastic and healthy adrenal tissue. Another patient (patient 1) showed a copy number variation of uncertain significance, involving a region 1.7 Mb on 8q21.3q22.1, not involving OMIM genes.

**Table 4:** Genetic finding: TP53 mutations features in tested patients.

**#**	**Exon**	**Codon**	**Nucleotide mutation**	**Type of mutation**	**Amino acid change**	**Germline/Somatic**	**LFS association (according to ClinVar)**	**Family history**
#2	6	607	G>A	Missense	pVal203Met	Germline (maternal segregation)	Uncertain significance (19)	Negative
				Heterozygosis				
#3	5	538	G>A	Missense	pGlu180Lys	Germline (maternal segregation)	Likely pathogenic (20)	Breast cancer in maternal grandmother
				Heterozygosis				
#4	4	358	A>T	Nonsense	pLys120*	Germline	Pathogenic (21, 22)	Negative
				Heterozygosis		*DE NOVO*		
#7	5	455	C>T	Missense	pPro152Leu	Germline (maternal segregation)	Pathogenic (23)	Brain tumor (NOS) in maternal grandfather (50 years-old)
				Heterozygosis				
#8	7	742	C>T	Missense	p.Arg248Trp	Germline (paternal segregation)	Pathogenic (23, 24)	Alveolar rhabdomyosarcoma in her brother (2 years old)
				Heterozygosis				
#13	5	472	G>A	Missense Heterozygosis	pVal143Met	Germline (maternal segregation)	Pathogenic (19, 25)	Choroid plexus carcinoma, neuroblastoma, soft tissue sarcoma and high-grade glioma in the maternal branch (see Figure 2)

## Discussion

Pediatric ACTs are very rare endocrine tumors in childhood with a highly heterogeneous and challenging prognosis. Recognized independent prognostic factors are older age (3, 10) and metastasis at the time of diagnosis (3), which in some cases could be attributed to delayed diagnosis. Our cohort is characterized by an excellent prognosis on long-term follow-up (media *n* = 5 years). Indeed, at present 12 patients are alive with no evidence of disease, and one is alive with evidence of metastatic disease. The 93% of patients were aged <4 years at diagnosis and with relative short time from symptoms onset (Table 1). Three patients were diagnosed in the course of other care, one prenatally and the other two through echographic evaluation for neonatal screening or urinary tract infection.

Routine prenatal ultrasound examinations have increased the detection of fetal tumors; some specific imaging features together with magnetic resonance imaging may help in the differential diagnosis as other common fetal abnormalities can sometimes mimic fetal tumors (26). This is very important for appropriate prenatal management of pregnancy and delivery in order to facilitate prompt postnatal treatment (26). Similarly, ultrasound screening in pediatric population can be used to reveal lesions like tumors or other pathologies of developmental age that are undetectable by clinical examination, before the onset of clinical symptoms (27, 28). This is particularly appropriate for patients with cancer predisposition, for example, in children with BWS (27, 29).

Although adult ACCs are classified following Weiss score, Ki67 > 10% and European Network for the Study of Adrenal Tumors for tumor stage (17), there are no clear pathological malignancy criteria for pediatric patients. Higher mitotic rate, higher percent of necrosis, and larger tumor size are usually associated with aggressive behavior (3). The Wieneke criteria, which include tumor size, local invasion, and histological features, have been reported useful in pediatric ACT malignancy definition and prognosis prediction (11, 18, 30). Recently, Picard and colleagues (31) proposed a pathological scoring system incorporating the Ki67 index ≥15% in a prognostication algorithm to guide adjuvant treatment in pediatric ACTs, mostly for those with incomplete resection. In our cohort, the Wieneke score could not predict clinical outcomes in patients who experienced metastatic disease.

Treatment of pediatric ACTs is often based on the results of adult studies, and the same guidelines are applied (6). When achievable, radical surgery remains the only successful treatment strategy. Capsule rupture with consequent tumor spreading, however, can be a frequent complication due to the tumor friability, mostly during laparoscopic resection. Thus, adrenalectomy in laparotomy is considered the standard of care (6, 32). We were able to perform surgery in all of our patients; open laparotomy was the preferred choice. Laparoscopy was used in one case by the neonatal surgeon for the antenatal diagnosed lesion, given the suspicion of a benign adrenal tumor. Careful follow-up with clinical, radiographic, and endocrine evaluation is mandatory after surgery to detect recurrence and metastasis early.

Adjuvant therapies for ACC have not been successful (6). Both radiation and chemotherapy are poorly effective, and the role of mitotane is not completely clear. Mitotane is a derivative of the insecticide dichlorodiphenyltricholorethane and has been used for treating ACC for more than five decades, also in association with chemotherapy (18, 33). It is the only drug approved for ACC by the US Food and Drug Administration, characterized by low efficacy rate and a narrow therapeutic window, which often involves serious toxicity (34, 35). Current evidence highlighted by a comprehensive review indicates that adjuvant mitotane significantly reduced the recurrence rate and mortality after surgery in nonmetastatic ACC patients (13, 18, 32). In our cohort, mitotane-based adjuvant therapy was administered for 12 months in seven patients with an acceptable tolerability and quality of life.

Despite the known ACC radioresistance, adjuvant radiotherapy of the tumor bed has been proposed and recommended in adult patients with microscopically incomplete resection (17, 18, 36). In pediatric population, radiation therapy has not been investigated for the high probability for patients of carrying germline *TP53* mutations and thus should be avoided.

No effective therapy is currently available for advanced and metastatic ACC; the only treatment allowing cure and long-term survival remains complete surgical resection (12, 37). Systemic chemotherapy and mitotane therapy are considered valuable therapeutic options in the treatment of advanced pediatric ACC patients (6, 38–40). Duration of mitotane treatment longer than 6 months and mitotane levels >14 mg/L were found to be associated with significantly better survival (38). The FIRM-ACT trial was conducted to determine whether treatment with etoposide, doxorubicin, cisplatin, and mitotane (EDP/M) prolonged survival as compared to streptozotocin and mitotane (Sz/M) in patients with inoperable advanced ACC. Rates of response and progression-free survival were significantly better with EDP plus mitotane as first-line therapy, with similar rates of toxic events [58%), but no significant differences in OS were observed (12)]. In our experience, one of the three patients who experienced metastatic disease obtained complete remission with platinum-based chemotherapy and mitotane. Overexpression of the IGF2 and IGF1R genes was described in ACT also in the pediatric setting (41), but trials testing the utility of insulin like growth factor receptor 1 inhibitors (e.g., linsitinib) have failed to provide advantage for adulthood ACC treatment (42).

Immunotherapy approaches have been recently investigated for this disease. In advanced ACC, pembrolizumab showed a significant and durable antitumor activity with a manageable safety profile (43–45). In the recent interim analysis of the phases 1–2 study, KEYNOTE-051 conducted in the pediatric setting, two of four patients with ACC showed partial responses to pembrolizumab therapy (46). In our cohort, two patients were treated by immunotherapy. Patient 8 showed early progressive disease. Patient 11 obtained durable complete remission after 24 months of pembrolizumab therapy (Figure 1). She is alive in not-evident disease after 3 years of follow-up.

Most childhood ACCs are reported in the context of cancer predisposition syndromes, in particular the Carney complex (CNC), the BWS, and the LFS. CNC, mostly due to germline inactivating mutations of PRKAR1A, is rarely associated with ACC but is the main cause of primary pigmented nodular adrenal diseases and usually linked to other tumors (somatotroph pituitary adenomas, thyroid, breast, and bone tumors, Sertoli tumors, melanocytic schwannoma, and cardiac and cutaneous myxomas) (5).

BWS is an overgrowth and tumor predisposition syndrome caused by genetic or epigenetic changes at the 11p15 locus. Childhood ACCs, together with embryonal tumors, represent the standard tumor spectrum of BWS (5). In our case studies, one patient was first clinically diagnosed with BWS, due to macrosomia, hyperinsulinism, hypoglycemia, and tumor at 1 month old. Then, the diagnosis of mosaic BWS was genetically confirmed by the evidence of chromosome 11 trisomy on healthy and neoplastic adrenal tissue but not on peripheral lymphocytes. Notably, this patient developed metastatic disease 3 months after surgery, treated by chemotherapy and mitotane, obtaining a complete remission with a 7-year follow-up.

LFS is a dramatic cancer predisposition syndrome, caused by germline inactivating mutations of *TP53* that highly expose to various and precocious cancer risk. Among the most common tumors in LFS are premenopausal breast cancer, soft-tissue sarcoma, osteosarcoma, central nervous system tumors, and ACC, the latter accounting for the 50–80% of pediatric cases. We found *TP53* mutation in 75% of tested patients (6/8) underlining the need to predict carrier and familial disease penetrance with potentially broad implications for clinical surveillance and counseling. Of note, the familial history was positive for cancer in four patients with *TP53* mutation and highly suggestive of LFS in two cases for the tumor histotypes and the very young age of the affected individuals. The most part of detected mutations were indeed already recognized as pathogenic (Table 3). In particular, the R248W missense *TP53* mutant that we found in patient 8 has been described to gain novel oncogenic activities (23, 26, 47). Interestingly, Pinto et al. (48) have investigated the clinicopathologic characteristics and outcomes of children with ACT without germline TP53 mutations. They found overlapping features with those reported for children with germline TP53 mutations, highlighting the central role of genetic or epigenetic alterations on chromosome 11p15 in pediatric ACT (48).

## Conclusion

Our experience with an ACT patient cohort of very young patients (12/13 aged <4 years at diagnosis), with relative short time from symptoms onset and localized disease at diagnosis, suggests an excellent prognosis with appropriate and aggressive diagnosis, staging, and surgical treatment. Our experience confirms age and metastasis as independent prognostic factors and the importance of early diagnosis, supported by already recommended echographic screening in neonates. In our patients, use of the Wieneke index, which is reported to be most accurate in predicting clinical outcomes in younger children, could not predict clinical outcomes.

We were able to treat all patients with surgery. Adjuvant mitotane was offered to 7 of 13 patients for 12 months with acceptable tolerance and no disease recurrence during therapy. In patients who developed metastatic disease, both immunotherapy and chemotherapy led to disease remission or control.

*TP53* mutation was found in 75% of tested patients confirming the need to perform genetic tests and familial counseling in this disease.

## Data Availability Statement

The raw data supporting the conclusions of this article will be made available by the authors, without undue reservation.

## Informed Consent

The authors declare that written informed consent was obtained from the patients' parents.

## Ethics Statement

The studies involving human participants were reviewed and approved by Bambino Gesù Children's Hospital Ethical Committee. Written informed consent to participate in this study was provided by the participants' legal guardian/next of kin. Written informed consent was obtained from the minor(s)' legal guardian/next of kin for the publication of any potentially identifiable images or data included in this article.

## Author Contributions

EM: conception (ideation), design of the work, structuration, acquisition of the data, writing, revision, and final approval to be published. ADG: structuration and interpretation of the data ACro: surgery acquisition of the data and revision. RC, AS, ACac, ACas, and MC: patients' management and revision. MDP: patients' management, structuration, and interpretation of the data. All authors contributed to the article and approved the submitted version.

## Conflict of Interest

The authors declare that the research was conducted in the absence of any commercial or financial relationships that could be construed as a potential conflict of interest.
